# Supplementary material for: A protocol for enhancing the diagnostic accuracy and predictive validity of neonatal opioid withdrawal syndrome: The utility of non-invasive clinical markers
Source: PLoS One. 2024 Sep 10;19(9):e0306176. doi: 10.1371/journal.pone.0306176 (PMC11386476; doi:10.1371/journal.pone.0306176)
Supplement: S1 Text — (DOCX) [file pone.0306176.s001.docx]

**Supplemental Materials**

**Supplemental Methods**

***Participants***

**Recruitment and Retention.** The study coordinator at Women and Infants Hospital reviews medical records daily to determine eligibility for the study and to recruit families prenatally using current established protocols. Recruitment in Utah also occurs at a prenatal specialty clinic that provides services for pregnant and postpartum birthing parents who are actively using substances or are in recovery for any addiction. In both sites, we follow a multi-step plan for recruitment and participation, described in Table B. We also use an evidence-based retention plan that was developed by our own experiences conducting research with high-risk families (Table C). Retention at the birth visit is supported by hospital protocols that require newborns with prenatal opioid exposure to remain in the hospital for at least 96 hours to monitor for NOWS. Retention at later ages is managed by project coordinators who engage in regular participant contact over the course of the study.

***Medical Record Abstraction***

Given that medical record abstraction often has a high error rate (1), at both sites, we followed a high-level framework based on engineering control theory (2). This framework posits that feedback or re-abstraction will help support abstracted data accuracy and mitigate any poor data quality within the medical records (i.e., data quality not controllable by the abstractor; Table E).

***Site-level variations***

Although the study protocol was consistent between sites, the neonatal care of opioid exposed infants may differ between hospitals. For example, researchers have found variability between sites in the threshold scores used to guide pharmacological treatment, the maximum dose and final dose of morphine (or other medications to treat NOWS), use of secondary medications, and length of treatment and the length of hospital stay. Therefore, we examine site-level difference in these areas of infant care.

**18 months (Time 3)**

***Infant Measures***

**Executive Functioning.** For the A-not-B task (3), infants are tasked with locating a hidden bouncy ball in one of two alternating locations, testing their ability to retain information about the ball’s location (working memory), while inhibiting their response to reach toward the previously rewarded location. The experimenter plays with a bouncy ball, showing it to the child and then hides the ball underneath the cup in the center of the table. The experimenter then moves the cup to side A (counterbalanced left/right), and then places a blank foam board in front of the cup obscuring the cup from the child’s view for 5 seconds. Simultaneously, the experimenter places another cup on side B. Then the experimenter removes the barrier and asks the child to point to where the ball is hidden (e.g., “Where’s the ball?”). If the child gets two correct guesses on side A, a reversal trial is presented next on side B. As the infant successfully completes the trials, the barrier is left up longer (5 seconds, 7 seconds, 10 seconds, etc.). Scores are calculated by number of correct responses out of total responses.

In the Wand task, the experimenter plays with a glitter wand for a few seconds, then places the wand in front of the child while telling the child not to touch the wand (e.g., “Now, *child’s name*, don’t touch. Don’t touch the wand”). If child grabs wand and picks it up then the task ends, and the experimenter gives the child permission to touch the want (e.g., “It’s okay; you can touch it now.”) If the child lightly touches then withdraws hand, the time keeps going until 30s is reached so that variation in behavior can be coded. After 30s, the experimenter ends the task and giver the child permission to touch the wand. Scores consist of latency to touch and variations of touch.

The Hide the Pots task consists of a snack placed under one of three opaque, colored pots. There are three trials in the practice phase, during which the child is asked directly to retrieve the snack from its hiding place (e.g., “Can you find the pot that has the snack inside?”). In the next phase, the snack is placed under one of the pots, but before the child can retrieve it, the pots are covered by a small blanket. The child must remember the location of the snack, remove the blanket, and then grab for where the snack was hidden. Toddlers received a 0 for incorrect grabs and a 1 for correct grabs on the first try.

**Supplemental Data Analysis**

**Sample Size**

A sample of 312 birthing parents and infants afforded sufficient power for the proposed analyses. For Aim 1, both the logistic and normally distributed regressions have adequate power (> 80% at α = .05) for continuous predictors with small to medium effect sizes. For the logistic regression, we estimated that 60% of newborns would receive a NOWS diagnosis. Assuming this proportion of diagnoses, for a continuous predictor (e.g., NNNS-II, cry, and sleep measures) the minimum effect that can be detected with 80% power is an odds ratio of 1.38. For the normally distributed regression (NOWS severity), the minimum effect that can be detected with 80% power for a continuous predictor is a Pearson’s correlation of +/-0.16. Missing data rates are expected to be very low for the newborn NOWS assessment, so no missing data were assumed.

For Aim 2, the proposed sample provides adequate power (> 80% at α = .05) for continuous predictors and a continuous-by-binary interaction for small to medium effects. These power analyses assumed 20% missing data, based on our prior studies with children with prenatal substance exposure (4,5). The minimum effects that can be detected with 80% power are 1) a Pearson’s correlation of +/-0.18 for a continuous main effect, 2) a Cohen’s *d* of 0.37 for a dichotomous main effect (60% NOWs diagnosis), and 3) a partial η^2^ of 0.04 for a continuous-by-dichotomous interaction.

**Preliminary Data Analyses**

The substantial number of predictors and the large sample size necessitates the use of consolidated measures, which improve the measurement properties of the predictors and dramatically reduce the number of statistical tests required, thus helping to control the Type 1 error rate. Since the NNNS-II exam provides 8 summary scores (see Table D) we model the heterogeneity of NNNS-II summary scores within the data using latent profile analysis (6,7), which will group infants into profiles based on their newborn neurobehavioral exam at birth. To determine the number of latent profiles, we compare goodness of fit indices using standard fit statistics (8–13).

The cry data requires additional analyses before incorporation into the primary analyses, based on the measures of loudness, voice pitch, formants timing, voicing and tension, or compression in the vocal tract. We conduct principal components analyses (PCA) to reduce several variables into a set of components that capture a significant proportion of variance in the cry indicators.

Exposure to prenatal opioid and other substances as measured in maternal hair at birth results in 17 different possible exposures. We include type of opioid exposure in our analyses along with prenatal exposure to nicotine, methamphetamine, cocaine, marijuana, and benzodiazepines, as predictors of NOWS onset and severity. We also examine whether trimester of exposure is predictive of NOWS diagnosis and severity. We use confirmatory factor analysis to test fit of our sociodemographic measures.

For consolidated measures demonstrating evidence for predicting NOWs diagnosis and severity, we consider exploratory analyses of the contributing components (e.g., specific drugs or sociodemographic measures). With these follow-up tests we utilize leave-one-out cross-validation to provide insight as to how robust the effects of individual indicators are given the limited sample, and how results generalize to an independent sample.

**Missing Data**

An incentive program is used to minimize missing data. Nonetheless, missing observations are anticipated at the 6- and 18-month follow-up visits. Modern methods for addressing missing data, such as multiple imputation, and full information maximum likelihood will be used to avoid the costly reductions in power and biased inferences that can occur with missing data. The effectiveness of both methods is contingent on identification of variables related to missing values, or the probability of a missing observation. Demographic variables, prior observations on study variables, and reasons for study discontinuation will be used in the application of modern missing data approaches to help ensure adequate power and unbiased inferences.

**Table A.** Participant inclusion and exclusion criteria.

| **Inclusion** | **Exclusion** |
| --- | --- |
| Birthing parents who used opioids during pregnancy identified prenatally or at birth via medical records  Maternal age of ≥18 years  Singleton pregnancy  Newborn is medically stable | Newborns with congenital abnormalities, genetic syndromes, persistent metabolic disturbances, or serious medical illnesses (e.g., sepsis, asphyxia, seizures, respiratory failure  Newborns unable to take oral medications  Caregiver unable to provide informed consent |

**Table B.** Multi-site recruitment plan.

| **Steps** | **Examples** |
| --- | --- |
| 1. Engaging participants in the research | We communicate with participants about the purpose of the research, which is to learn more about the development of children with prenatal opioid exposure. We describe the importance of conducting longitudinal research, and that we want to learn more about the factors that help children thrive as they develop. |
| 2a. Benefiting participants: payment | Participants report that they appreciate grocery store gift cards. Therefore, all payments are in the form of grocery store gift cards. Participants are paid $50 at the hospital, $75 at 6 months, and $100 at 18 months. |
| 2b. Benefiting participants: Advancing science, community, and child health | Participants reported that they chose to participate in our study to benefit other birthing parents and babies. Therefore, recruitment coordinators engage participants in a conversation about how their participation advances the science of child development in children with prenatal opioid exposure. Their participation also helps the community by learning more about the factors that promote positive child health outcomes. Finally, they learn more about how their own child is growing and developing. |
| 3. Removing barriers to participation | We developed a Frequently Asked Questions sheet to address potential concerns. Recruitment coordinators build trust with participants by explaining that data are confidential. Recruitment coordinators are trained and monitored to ensure that they do not discriminate against participants because they are using substances. Recruitment coordinators clearly describe how the results will be used: De-identified data are used in journal publications and conference presentations to better understand how to promote child health. We also provide transportation to participants and provide them with flexible days and times for the study visit, and childcare as needed. If visits are scheduled in the evening, we provide dinner for the families. |
| 4. Developing clear informational materials | We developed clear and simple informational materials that describe the purpose of the study and study procedures to aid in recruitment. We provide health information resources from NIH for participants to learn more about prenatal substance exposure. |

**Table C.** Multi-site retention plan.

| **Steps** | **Examples** |
| --- | --- |
| 1. Paying participants | Participants are paid $50 at the hospital, $75 at 6 months, and $100 at 18 months. All payments are in the form of grocery store gift cards. |
| 2. Maintaining participant contact | Participants are mailed thank-you cards after each visit. The child is mailed a birthday card each year. Mother’s Day cards are sent each year. Every 6 months, a newsletter is mailed containing evidence-based methods for promoting healthy child development from the organization Zero to Three (see example newsletter here: https://canlab.psych.utah.edu/for-parents.php). |
| 3. Communicating results of the research study | The ECHO study found that the number one reason why a participant left a research study is because no results were communicated. As soon as possible, we mail participants a newsletter that uses data from our study to highlight how participants can promote positive child health outcomes. |
| 4. Maintaining project coordinators in both sites who develop rapport with families | In our research we have found that a single project coordinator who develops a relationship with families is critical for retaining participants. We ensure as much as possible that our project coordinators (one for each site) can work for the duration of the study, and ideally beyond. These project coordinators obtain several phone numbers, email addresses, and get permission to approach participants on social media to ensure high retention. |
| 5. Communicating our long-term commitment to participants and the community | In our newsletters we detail our commitment to learning more about promoting the health and well-being of children with prenatal substance exposure. We continue to submit grants to obtain long-term follow-up data. |
| 6. Monitoring staff training and performance | Given that staff training directly impacts recruitment and retention, we work to reduce staff turnover, conduct regular trainings, and maintain strong rapport among staff members. We reduce staff turnover by meeting with staff twice per year to hear their concerns and their sense of fulfillment in their roles. We conduct trainings at least twice per year for new staff and to ensure proper data collection procedures. We maintain strong rapport among staff by hosting social events and provide staff with food and outings (e.g., bowling nights, pizza parties). |

**Table D.** NeoNatal Neurobehavioral Scale (NNNS-II).

| **NNNS-II Summary Scales** | **Description** |
| --- | --- |
| Attention | Infant response to experimenter stimuli indicated by appropriate head turning, gaze, and sustained alertness |
| Handling | Score based on the strategies the experimenter used during orientation tasks to keep the infant in an alert state |
| Quality of Movement | Measure of smooth infant control of motor, activity lacking startles, tremors, and jitters |
| Regulation | A broad scale based on the infant's ability to coordinate their movements, physiology, & state; also incorporates the infant's ability to be soothed and their responses to the experimenter's cuddling and consoling |
| Non-optimal Reflexes | Reflex responses from the infant that are weaker or stronger than what is optimal or expected |
| Stress/Abstinence | Number of observed infant stress/abstinence signs across various organ systems |
| Arousal | Infant's level of motor activity in response to handling, irritability, and fussiness during the examination |
| Tone | Increased/decreased muscle rigidity and tone |

**Table E.** Medical record abstraction best practices.

| Medical Record Abstraction Protocol |
| --- |
| *Abstractor training* |
| • Components of abstractor training: |
| a.) Overview of the study |
| b.) How data are collected |
| c.) Abstraction specifications, i.e., definitions, guidelines, and conventions |
| d.) Training on the abstraction form in REDCap |
| e.) Practice abstractions with feedback |
| f.) Examples of difficult cases |
| *Abstracting environment* |
| • Positive relationships with medical staff |
| • Ongoing communication among abstractors, e.g., to discuss difficult cases |
| • Minimal interruptions and time pressures during abstraction |
| • Easy access to medical records through Epic healthcare software |
| *Abstraction process* |
| • Relevant parts of the medical record are reviewed before abstracting |
| • Data are abstracted into computerized forms on REDCap |
| • Computerized error checks during data entry for missing, out-of-range, or illogical values |
| • Nonpharmacological abstraction may occur during the patient encounter |
| *Abstraction guidelines and definitions available in a codebook, protocol, and REDCap forms* |
| • Standardized abstraction process |
| • Specified data locations in the medical records |
| • Available synonyms and abbreviations |
| • Conventions for common problems, e.g., multiple values |
| • Guidelines for missing information, e.g., unknown value for characters |
| • Identification of less accurately abstracted data, e.g., weaning and medication dosage |
| • Minimized calculations and converting units, e.g., weaning (Utah) |
| • Avoidance of subjective data |
| • Inclusion and exclusion criteria |
| *Factors Inherent in Medical Records that Decrease the Accuracy of Abstracted Data* |
| • Error, inconsistency, or conflicting information, e.g., medication start time |
| • Missing information, e.g., nonpharmacological interventions |
| • Variability of assessment skills, e.g., night staff often overscores Finnegan |
| *Data Quality Control Activities: Re-abstraction* |
| • Independent re-abstraction of all cases by an experienced abstractor |
| • Review re-abstraction results, e.g., discrepancies or difficulty areas with abstractors |
| • Monitor abstractor performance |
