# Supplementary material for: A protocol for enhancing the diagnostic accuracy and predictive validity of neonatal opioid withdrawal syndrome: The utility of non-invasive clinical markers
Source: PLoS One. 2024 Sep 10;19(9):e0306176. doi: 10.1371/journal.pone.0306176 (PMC11386476; doi:10.1371/journal.pone.0306176)
Supplement: Attachment — Submitted filename: Review_12.20.23.docx [file pone.0306176.s002.docx]

PONE-D-23-04246

Enhancing the diagnostic accuracy and predictive validity of neonatal opioid withdrawal syndrome: The utility of non-invasive clinical markers

PLOS ONE

Dear Dr. Maylott,

Thank you for submitting your manuscript to PLOS ONE. After careful consideration, we feel that it has merit but does not fully meet PLOS ONE’s publication criteria as it currently stands. Therefore, we invite you to submit a revised version of the manuscript that addresses the points raised during the review process.


Please submit your revised manuscript by Dec 14 2023 11:59PM. If you will need more time than this to complete your revisions, please reply to this message or contact the journal office at [plosone@plos.org](mailto:plosone@plos.org). When you're ready to submit your revision, log on to [https://www.editorialmanager.com/pone/](https://urldefense.com/v3/__https:/www.editorialmanager.com/pone/__;!!OToaGQ!rb318e_jrBEUBZ5YXGdkT3xoJxWpXIMwW0OtUbrncjboKa3nltwu3DZ51vCQCNF1FvYy8OIsl_hKBflK3zf9$) and select the 'Submissions Needing Revision' folder to locate your manuscript file.

Please include the following items when submitting your revised manuscript:

- A rebuttal letter that responds to each point raised by the academic editor and reviewer(s). You should upload this letter as a separate file labeled 'Response to Reviewers'.
- A marked-up copy of your manuscript that highlights changes made to the original version. You should upload this as a separate file labeled 'Revised Manuscript with Track Changes'.
- An unmarked version of your revised paper without tracked changes. You should upload this as a separate file labeled 'Manuscript'.

If you would like to make changes to your financial disclosure, please include your updated statement in your cover letter. Guidelines for resubmitting your figure files are available below the reviewer comments at the end of this letter.

If applicable, we recommend that you deposit your laboratory protocols in protocols.io to enhance the reproducibility of your results. Protocols.io assigns your protocol its own identifier (DOI) so that it can be cited independently in the future. For instructions see: [https://journals.plos.org/plosone/s/submission-guidelines#loc-laboratory-protocols](https://urldefense.com/v3/__https:/journals.plos.org/plosone/s/submission-guidelines*loc-laboratory-protocols__;Iw!!OToaGQ!rb318e_jrBEUBZ5YXGdkT3xoJxWpXIMwW0OtUbrncjboKa3nltwu3DZ51vCQCNF1FvYy8OIsl_hKBY_cTfuT$). Additionally, PLOS ONE offers an option for publishing peer-reviewed Lab Protocol articles, which describe protocols hosted on protocols.io. Read more information on sharing protocols at [https://plos.org/protocols?utm_medium=editorial-email&utm_source=authorletters&utm_campaign=protocols](https://urldefense.com/v3/__https:/plos.org/protocols?utm_medium=editorial-email&utm_source=authorletters&utm_campaign=protocols__;!!OToaGQ!rb318e_jrBEUBZ5YXGdkT3xoJxWpXIMwW0OtUbrncjboKa3nltwu3DZ51vCQCNF1FvYy8OIsl_hKBVVaymZq$).

We look forward to receiving your revised manuscript.

Kind regards,

Ammal Mokhtar Metwally, Ph.D (MD)
Academic Editor
PLOS ONE

Journal Requirements:

When submitting your revision, we need you to address these additional requirements.

1. Please ensure that your manuscript meets PLOS ONE's style requirements, including those for file naming. The PLOS ONE style templates can be found at 
[https://journals.plos.org/plosone/s/file?id=wjVg/PLOSOne_formatting_sample_main_body.pdf](https://urldefense.com/v3/__https:/journals.plos.org/plosone/s/file?id=wjVg*PLOSOne_formatting_sample_main_body.pdf__;Lw!!OToaGQ!rb318e_jrBEUBZ5YXGdkT3xoJxWpXIMwW0OtUbrncjboKa3nltwu3DZ51vCQCNF1FvYy8OIsl_hKBR_G7sJ_$) and 
[https://journals.plos.org/plosone/s/file?id=ba62/PLOSOne_formatting_sample_title_authors_affiliations.pdf](https://urldefense.com/v3/__https:/journals.plos.org/plosone/s/file?id=ba62*PLOSOne_formatting_sample_title_authors_affiliations.pdf__;Lw!!OToaGQ!rb318e_jrBEUBZ5YXGdkT3xoJxWpXIMwW0OtUbrncjboKa3nltwu3DZ51vCQCNF1FvYy8OIsl_hKBTW3OuD3$)

2. Please include “Protocol” in the manuscript title.

Authors: “Protocol” was added to the title: A protocol for enhancing the diagnostic accuracy and predictive validity of neonatal opioid withdrawal syndrome: The utility of non-invasive clinical markers

3. We note that you have indicated that data from this study are available upon request. PLOS only allows data to be available upon request if there are legal or ethical restrictions on sharing data publicly. For more information on unacceptable data access restrictions, please see [http://journals.plos.org/plosone/s/data-availability#loc-unacceptable-data-access-restrictions.](https://urldefense.com/v3/__http:/journals.plos.org/plosone/s/data-availability*loc-unacceptable-data-access-restrictions.__;Iw!!OToaGQ!rb318e_jrBEUBZ5YXGdkT3xoJxWpXIMwW0OtUbrncjboKa3nltwu3DZ51vCQCNF1FvYy8OIsl_hKBaIZK63X$)

Authors: Because data collection is currently ongoing and no data was used in this protocol paper, no data is currently available to be shared. We have stated this in the Data Availability section:

**“Data Availability:** As data collection for the present study protocol is ongoing, no study data is currently available. Deidentified research data will be made available upon reasonable request when the study is completed and published.”

In your revised cover letter, please address the following prompts:

a) If there are ethical or legal restrictions on sharing a de-identified data set, please explain them in detail (e.g., data contain potentially sensitive information, data are owned by a third-party organization, etc.) and who has imposed them (e.g., an ethics committee). Please also provide contact information for a data access committee, ethics committee, or other institutional body to which data requests may be sent.

b) If there are no restrictions, please upload the minimal anonymized data set necessary to replicate your study findings as either Supporting Information files or to a stable, public repository and provide us with the relevant URLs, DOIs, or accession numbers. For a list of acceptable repositories, please see [http://journals.plos.org/plosone/s/data-availability#loc-recommended-repositories](https://urldefense.com/v3/__http:/journals.plos.org/plosone/s/data-availability*loc-recommended-repositories__;Iw!!OToaGQ!rb318e_jrBEUBZ5YXGdkT3xoJxWpXIMwW0OtUbrncjboKa3nltwu3DZ51vCQCNF1FvYy8OIsl_hKBTv3BRTg$).

We will update your Data Availability statement on your behalf to reflect the information you provide.

4. We note that you have referenced (Unpublished) om page 28, which has currently not yet been accepted for publication. Please remove this from your References and amend this to state in the body of your manuscript: (ie “Bewick et al. [Unpublished]”) as detailed online in our guide for authors
[http://journals.plos.org/plosone/s/submission-guidelines#loc-reference-style](https://urldefense.com/v3/__http:/journals.plos.org/plosone/s/submission-guidelines*loc-reference-style__;Iw!!OToaGQ!rb318e_jrBEUBZ5YXGdkT3xoJxWpXIMwW0OtUbrncjboKa3nltwu3DZ51vCQCNF1FvYy8OIsl_hKBaNTssvN$)

 Authors: We added i.e., Weiss et al. [Unpublished] to the body of the text.

Additional Editor Comments:

The manuscript is interested meanwhile, the reviewers have raised a number of points which we believe would improve the manuscript and may allow a revised version to be published in PLOS one.


[Note: HTML markup is below. Please do not edit.]

Reviewers' comments:

Reviewer's Responses to Questions

**Comments to the Author**

1. Does the manuscript provide a valid rationale for the proposed study, with clearly identified and justified research questions?

The research question outlined is expected to address a valid academic problem or topic and contribute to the base of knowledge in the field.

Reviewer #1: Yes

Reviewer #2: Yes

2. Is the protocol technically sound and planned in a manner that will lead to a meaningful outcome and allow testing the stated hypotheses?

The manuscript should describe the methods in sufficient detail to prevent undisclosed flexibility in the experimental procedure or analysis pipeline, including sufficient outcome-neutral conditions (e.g. necessary controls, absence of floor or ceiling effects) to test the proposed hypotheses and a statistical power analysis where applicable. As there may be aspects of the methodology and analysis which can only be refined once the work is undertaken, authors should outline potential assumptions and explicitly describe what aspects of the proposed analyses, if any, are exploratory.

Reviewer #1: Yes

Reviewer #2: Yes

3. Is the methodology feasible and described in sufficient detail to allow the work to be replicable?


Descriptions of methods and materials in the protocol should be reported in sufficient detail for another researcher to reproduce all experiments and analyses. The protocol should describe the appropriate controls, sample size calculations, and replication needed to ensure that the data are robust and reproducible.

Reviewer #1: Yes

Reviewer #2: Yes

4. Have the authors described where all data underlying the findings will be made available when the study is complete?

The [PLOS Data policy](https://urldefense.com/v3/__https:/journals.plos.org/plosone/s/materials-and-software-sharing__;!!OToaGQ!rb318e_jrBEUBZ5YXGdkT3xoJxWpXIMwW0OtUbrncjboKa3nltwu3DZ51vCQCNF1FvYy8OIsl_hKBUfanxZA$) requires authors to make all data underlying the findings described in their manuscript fully available without restriction, with rare exception, at the time of publication. The data should be provided as part of the manuscript or its supporting information, or deposited to a public repository. For example, in addition to summary statistics, the data points behind means, medians and variance measures should be available. If there are restrictions on publicly sharing data—e.g. participant privacy or use of data from a third party—those must be specified.

Reviewer #1: No

Reviewer #2: Yes

5. Is the manuscript presented in an intelligible fashion and written in standard English?

PLOS ONE does not copyedit accepted manuscripts, so the language in submitted articles must be clear, correct, and unambiguous. Any typographical or grammatical errors should be corrected at revision, so please note any specific errors here.

Reviewer #1: Yes

Reviewer #2: Yes

6. Review Comments to the Author

Please use the space provided to explain your answers to the questions above and, if applicable, provide comments about issues authors must address before this protocol can be accepted for publication. You may also include additional comments for the author, including concerns about research or publication ethics.

You may also provide optional suggestions and comments to authors that they might find helpful in planning their study.

(Please upload your review as an attachment if it exceeds 20,000 characters)

Reviewer #1: On line 37, the word "consent" was left out and should be added.

Authors: We thank the reviewer for pointing out this error. We added “consent” to line 37.

Paragraph beginning line 79 - the authors should clarify that the mu-receptor binding, and then lack of, is primarily responsible for the symptoms of withdrawal as described; individuals not familiar with this area may not make that connection.

Authors: We now clarify this connection for the reader:

“Consequently, the μ-receptor binding of opioids and then the cessation binding (i.e., the cessation of opioid exposure) after birth may lead to withdrawal signs, including autonomic instability, high-pitched cry, irritability, tremors, and difficulty feeding and sleeping (12).”

Line 95 - May want to change the wording "evidenced-based". Given the recent ongoing studies evaluating the assessment of infants with NOWS, this is now becoming evidenced-based. Would delete that phrase.

Authors: We changed the wording “evidenced-based” on line 95 to describe the limitations of the current clinical assessments in more detail:

“However, these diagnostic tools are not often thoroughly researched, psychometrically sound, and/or standardized, and the choice of diagnostic tool depends on independent hospital protocols (15).”

Line 106 authors state NNNS-II; later it is called NNNS. Please be consistent.

Authors: We now refer to the NeoNatal Neurobehavioral Scale as the NNNS-II, based off the original NNNS exam.

The authors report that infants exposed to both opioids and cocaine have louder and higher pitched cries (line 133). How can cry be used in infants with co-exposures? Is this a limitation?

Authors: We discuss the implications of exposures and co-exposures for infant cry measures:

“Infants with *in utero* opioid exposure tend to have shorter cry utterances and extremely high-pitched cries, and infants co-exposed to opioids and cocaine have comparably louder and higher pitched cries (20). High-pitched and hyperphonated cries have been reported in infants with neurologic problems (21), suggesting that infants with substance exposures, especially those with polysubstance exposures may be at increased neurological risk.”

The authors report that maternal stress can negatively impact fetal development, and thus impact the cortisol levels. How does the maternal stress play into the impact on HRV? That should be considered when assessing the HRV and may limit the applicability of HRV in certain populations.

Similarly, including maternal stressors as a confounding factor in the cortisol measurements is important.

Authors: We thank the reviewer for this feedback. We added a limitations section to discuss maternal stress as a confounding variable:

“This study is not without limitations. Confounding factors like maternal stress—due to poverty, psychopathology, and poor general health, as well as exposure to violence, trauma, and/or abuse—may complicate the interpretations of our findings. For example, while fetal and newborn heart rate variability have been linked to opioid exposure (30,31), greater endorsements of prenatal stress have also been associated with reduced fetal heart rate variability (92). Similarly maternal stress may also play a role in infant cortisol levels (33,36). Therefore, we measure maternal sociodemographic risk, psychopathology, and trauma history in this protocol to account for potential confounding.”

In assessing the COMT gene - has this also been assessed in individuals with higher stress levels to ensure that the increase in truly in relation to the opiate exposure?

Authors: The COMT gene has been assessed in individuals with higher stress exposure. At present it is unclear whether associations between opioid exposure and COMT gene is due to opioid exposure or related exposures such as stress. We included this as a limitation:

“Further, the *COMT* and *NR3C1* gene methylation are also related to both infant stress and opioid exposure, which may confound findings.”

The authors conclude in lines 217-220 that infants with a longer gestation have worse NOWS. This should be reconsidered. Currently, the assessments that are used are focused on the term infant and what is expected at that gestational age. This then leads to the inability to accurately assess preterm infants for NOWS, given that they have very different neurological development at birth and during the period of expected withdrawal. I would not conclude that term infants have worse withdrawal, but rather that we are not able to assess withdrawal in preterm accurately.

Authors: We thank the reviewer for this suggestion. We added this an explanation for late term infants’ likelihood of being treated for NOWS:

“These findings may be due to the high permeability of the placenta in the late third trimester (50) or that current assessments are not geared toward assessing NOWS in preterm infants who may display differences in neurological development and, therefore, withdrawal signs at birth.”

Line 262 - states that data is collected 24 hours of life, when infant is pre-symptomatic. I would take out pre-symptomatic, as some infants do develop withdrawal symptoms at that time, especially when they are exposed to opioids with short half-lives.

Authors: We thank the reviewer for highlighting this point. We removed “pre-symptomatic” as a descriptive as some of our infants may show symptoms prior to the 24-hour mark.

What are "caregiver transitions"? This should be defined.

Authors: We now define caregiver transitions:

“We record the number of primary caregiver changes over the course of the study, starting at birth. This occurs when there is a change in custody. The child’s primary caregiver at birth is the biological mother. Caregiver transitions occur when the child is placed with a foster parent, adoptive parent, or other caregiver (e.g., family member). Reasons for caregiver transitions include DCFS involvement (e.g., neglect, abuse, substance use), incarceration of caregiver, death of caregiver, adoption, and/or reunification, among others.”

Does the removal of the HRV monitor result in stress in the infant that could impact the samples collected for cortisol / gene measurement?

Authors: We now clarify that the removal of the HRV monitor does not impact the samples collected as we remove the ECG leads and wires after the saliva and buccal samples have been collected:

“ECG leads and wires are removed post newborn assessments, so that we cause no additional stress during the exams.”

Please include citations to support line 283 - the family resource scale has high test-retest reliability and predictive validity.

Authors: We thank the authors for pointing out this discrepancy. We report the citation to verify reliability and predictive validity of the Family Resource Scale

61.	Dunst CJ, Leet HE. Family Resource Scale. Child Care Health Dev [Internet]. 1987; Available from: https://doi.org/10.1037/t33262-000

If the pregnant individual does not have long enough hair, are they excluded from the study?

Authors: We retained all participants even if they were not able to provide hair samples:

“Finally, we collect maternal hair samples for toxicology analyses. If the hair is too short or birthing parents refuse to provide the hair sample, the missing data is tracked but participants are still included in the study.”

Were all infants included in the study singleton? Term? Breastfed or bottle fed?

Authors: Our sample is not limited to singleton term infants and infants can be breast or bottle fed. We clarify this in the study population section:

“Newborns are medically stable and include singleton and twin pregnancies, term and preterm infants, and breast- and bottle-fed infants.”

What is the timing of this study (later it is stated 2020)? Why was Bayley III used instead of IV? The Bayley III would have been outdated at that time.

Authors: We thank the reviewer for highlighting this error. The study officially began in 2021 and the Bayley IV is used. We updated the appropriate sections in the paper.

The authors say "Infant measures" when describing the 18 month visit. The term infant is no longer applicable and should be changed.

Authors: We now refer to “Infant measures” at 18-months as “Toddler measures.”

If the cry analyzer is used in an open bay NICU, how accurate is it? Will it pick up the noise of surrounding infants?

Authors: We collect cry data in open bay NICUs and Intermediate Care Nurseries. After recordings are uploaded, they are screened and filtered for ambient noise so that only the target infant cries are included in analyses.

Are there results of this protocol? There is a lot of description of the protocol, with almost no discussion and no results. The title implies that there should be results given. If this is meant to be a protocol paper, that should be clearly stated in the text rather than simply stated as the article type.

Authors: We updated the title to include “protocol” to clarify there are no findings discussed within the manuscript.

The discussion should also include more points about confounding factors that may impact the assessments, strengths and weaknesses of the approach, etc.

Authors: We thank the reviewer for the feedback. We added potential confounding factors and discussed their potential impact on the study (page 22 & 23).

Reviewer #2: I read with inerest the paper by Mylott and colleagues about a possible protocol for the early identification of NOWS, aiming to provide a more targeted approach to neonates ar risk, allowing an early identification of NOWS and also of those infants that will not develop this problem. moreover the later timepoints will allow to evaluate the neurodeveoplment of this particular population, that is evidently at risk for neurodevelopment impairment.
The suggested protocol enclosed multiple approaches to target from different sides the problem, analysing different aspects of the same issue.
The proposal is surely interesting and complete, and the results of its application will consequently be awaited.

The main concern about this approach is obviously the complexity, that requires many different approaches and is consequently time-consuming and makes it less feasible in a setting with less resources. Authors should adress this aspect.

Authors: We thank the reviewer for the constructive feedback. We addressed the reviewers concerns about the number of approaches and feasibility in a high-risk and a hard to recruit/retain population on page 23 and in the supplemental methods and tables:

“The current study protocol recruits from a hard to retain population, and the length of the study and the number of study measures increase the complexity of the study, which may make recruitment and retention less feasible. However, to combat this limitation, we implement an evidence-based recruitment and retention plan for high-risk families (See Supplemental Methods and Tables S2 & S3).”

7. PLOS authors have the option to publish the peer review history of their article ([what does this mean?](https://urldefense.com/v3/__https:/journals.plos.org/plosone/s/editorial-and-peer-review-process*loc-peer-review-history__;Iw!!OToaGQ!rb318e_jrBEUBZ5YXGdkT3xoJxWpXIMwW0OtUbrncjboKa3nltwu3DZ51vCQCNF1FvYy8OIsl_hKBbGZsysT$)). If published, this will include your full peer review and any attached files.


If you choose “no”, your identity will remain anonymous but your review may still be made public.


**Do you want your identity to be public for this peer review?** For information about this choice, including consent withdrawal, please see our [Privacy Policy](https://urldefense.com/v3/__https:/www.plos.org/privacy-policy__;!!OToaGQ!rb318e_jrBEUBZ5YXGdkT3xoJxWpXIMwW0OtUbrncjboKa3nltwu3DZ51vCQCNF1FvYy8OIsl_hKBZ7qqqBy$).

Reviewer #1: No

Reviewer #2: No

[NOTE: If reviewer comments were submitted as an attachment file, they will be attached to this email and accessible via the submission site. Please log into your account, locate the manuscript record, and check for the action link "View Attachments". If this link does not appear, there are no attachment files.]

While revising your submission, please upload your figure files to the Preflight Analysis and Conversion Engine (PACE) digital diagnostic tool, [https://pacev2.apexcovantage.com/](https://urldefense.com/v3/__https:/pacev2.apexcovantage.com/__;!!OToaGQ!rb318e_jrBEUBZ5YXGdkT3xoJxWpXIMwW0OtUbrncjboKa3nltwu3DZ51vCQCNF1FvYy8OIsl_hKBUNoJLVX$). PACE helps ensure that figures meet PLOS requirements. To use PACE, you must first register as a user. Registration is free. Then, login and navigate to the UPLOAD tab, where you will find detailed instructions on how to use the tool. If you encounter any issues or have any questions when using PACE, please email PLOS at [figures@plos.org](mailto:figures@plos.org). Please note that Supporting Information files do not need this step.
