# Supplementary material for: A protocol for enhancing the diagnostic accuracy and predictive validity of neonatal opioid withdrawal syndrome: The utility of non-invasive clinical markers
Source: PLoS One. 2024 Sep 10;19(9):e0306176. doi: 10.1371/journal.pone.0306176 (PMC11386476; doi:10.1371/journal.pone.0306176)
Supplement: Attachment — Submitted filename: Review_2_4.14.24.docx [file pone.0306176.s003.docx]

PONE-D-23-04246R1

A protocol for enhancing the diagnostic accuracy and predictive validity of neonatal opioid withdrawal syndrome: The utility of non-invasive clinical markers

PLOS ONE

Dear Dr. Maylott,

Thank you for submitting your manuscript to PLOS ONE. After careful consideration, we feel that it has merit but does not fully meet PLOS ONE’s publication criteria as it currently stands. Therefore, we invite you to submit a revised version of the manuscript that addresses the points raised during the review process.

Please submit your revised manuscript by Apr 14 2024 11:59PM. If you will need more time than this to complete your revisions, please reply to this message or contact the journal office at [plosone@plos.org](mailto:plosone@plos.org). When you're ready to submit your revision, log on to [https://www.editorialmanager.com/pone/](https://urldefense.com/v3/__https:/www.editorialmanager.com/pone/__;!!OToaGQ!qPCw3_ZC9IXOckCyoGIyu8uNbaMpNvlu6HSo5mtqcWPut8o-UQc70dT8oMwdJ83V-Z5FlUGh1rd3UQw8zCvW$) and select the 'Submissions Needing Revision' folder to locate your manuscript file.

Please include the following items when submitting your revised manuscript:

- A rebuttal letter that responds to each point raised by the academic editor and reviewer(s). You should upload this letter as a separate file labeled 'Response to Reviewers'.
- A marked-up copy of your manuscript that highlights changes made to the original version. You should upload this as a separate file labeled 'Revised Manuscript with Track Changes'.
- An unmarked version of your revised paper without tracked changes. You should upload this as a separate file labeled 'Manuscript'.

If you would like to make changes to your financial disclosure, please include your updated statement in your cover letter. Guidelines for resubmitting your figure files are available below the reviewer comments at the end of this letter.

If applicable, we recommend that you deposit your laboratory protocols in protocols.io to enhance the reproducibility of your results. Protocols.io assigns your protocol its own identifier (DOI) so that it can be cited independently in the future. For instructions see: [https://journals.plos.org/plosone/s/submission-guidelines#loc-laboratory-protocols](https://urldefense.com/v3/__https:/journals.plos.org/plosone/s/submission-guidelines*loc-laboratory-protocols__;Iw!!OToaGQ!qPCw3_ZC9IXOckCyoGIyu8uNbaMpNvlu6HSo5mtqcWPut8o-UQc70dT8oMwdJ83V-Z5FlUGh1rd3UUjfXEAn$). Additionally, PLOS ONE offers an option for publishing peer-reviewed Lab Protocol articles, which describe protocols hosted on protocols.io. Read more information on sharing protocols at [https://plos.org/protocols?utm_medium=editorial-email&utm_source=authorletters&utm_campaign=protocols](https://urldefense.com/v3/__https:/plos.org/protocols?utm_medium=editorial-email&utm_source=authorletters&utm_campaign=protocols__;!!OToaGQ!qPCw3_ZC9IXOckCyoGIyu8uNbaMpNvlu6HSo5mtqcWPut8o-UQc70dT8oMwdJ83V-Z5FlUGh1rd3UZ0dJx2w$).

We look forward to receiving your revised manuscript.

Kind regards,

Ammal Mokhtar Metwally, Ph.D (MD)
Academic Editor
PLOS ONE

**Additional Editor Comments:**

The manuscript is interested meanwhile, the reviewers have raised a number of points which we believe would improve the manuscript and may allow a revised version to be published in PLOS one.


[Note: HTML markup is below. Please do not edit.]

Reviewers' comments:

Reviewer's Responses to Questions

**Comments to the Author**

1. Does the manuscript provide a valid rationale for the proposed study, with clearly identified and justified research questions?

The research question outlined is expected to address a valid academic problem or topic and contribute to the base of knowledge in the field.

Reviewer #1: Yes

Reviewer #3: Yes

2. Is the protocol technically sound and planned in a manner that will lead to a meaningful outcome and allow testing the stated hypotheses?

The manuscript should describe the methods in sufficient detail to prevent undisclosed flexibility in the experimental procedure or analysis pipeline, including sufficient outcome-neutral conditions (e.g. necessary controls, absence of floor or ceiling effects) to test the proposed hypotheses and a statistical power analysis where applicable. As there may be aspects of the methodology and analysis which can only be refined once the work is undertaken, authors should outline potential assumptions and explicitly describe what aspects of the proposed analyses, if any, are exploratory.

Reviewer #1: Yes

Reviewer #3: Yes

3. Is the methodology feasible and described in sufficient detail to allow the work to be replicable?


Descriptions of methods and materials in the protocol should be reported in sufficient detail for another researcher to reproduce all experiments and analyses. The protocol should describe the appropriate controls, sample size calculations, and replication needed to ensure that the data are robust and reproducible.

Reviewer #1: Yes

Reviewer #3: Yes

4. Have the authors described where all data underlying the findings will be made available when the study is complete?

The [PLOS Data policy](https://urldefense.com/v3/__https:/journals.plos.org/plosone/s/materials-and-software-sharing__;!!OToaGQ!qPCw3_ZC9IXOckCyoGIyu8uNbaMpNvlu6HSo5mtqcWPut8o-UQc70dT8oMwdJ83V-Z5FlUGh1rd3UblHr8wD$) requires authors to make all data underlying the findings described in their manuscript fully available without restriction, with rare exception, at the time of publication. The data should be provided as part of the manuscript or its supporting information, or deposited to a public repository. For example, in addition to summary statistics, the data points behind means, medians and variance measures should be available. If there are restrictions on publicly sharing data—e.g. participant privacy or use of data from a third party—those must be specified.

Reviewer #1: No

Reviewer #3: Yes

5. Is the manuscript presented in an intelligible fashion and written in standard English?

PLOS ONE does not copyedit accepted manuscripts, so the language in submitted articles must be clear, correct, and unambiguous. Any typographical or grammatical errors should be corrected at revision, so please note any specific errors here.

Reviewer #1: Yes

Reviewer #3: Yes

6. Review Comments to the Author

Please use the space provided to explain your answers to the questions above and, if applicable, provide comments about issues authors must address before this protocol can be accepted for publication. You may also include additional comments for the author, including concerns about research or publication ethics.

You may also provide optional suggestions and comments to authors that they might find helpful in planning their study.


(Please upload your review as an attachment if it exceeds 20,000 characters)

Reviewer #1:

Under clinical measure 2: citation should be added to line 138-139: "preliminary acoustic cry data showed that it was 81% accurate in predicting NOWS."

Authors: We thank the reviewer for pointing out this discrepancy. We reference preliminary data that is unpublished, so we added “(Unpublished data)” at the conclusion of the sentence:

“Preliminary acoustic cry data showed that it was 81% accurate in predicting NOWS; moreover, acoustics were more accurate in some cases when the observational crying score was incorrectly evaluated (Unpublished data).”

Lines 150-151: While the higher doses of opioid exposure show more disturbance in sleep, higher doses of opioid exposure are not necessarily linked to more severe withdrawal in the infant. Please revise this sentence.

Authors: We appreciate the feedback and agree that higher doses of opioid exposure do not necessarily result in more severe withdrawal; therefore, we changed “severity” to “neurobehavior” as outlined below:

“Further, newborns exposed to higher doses of opioids *in utero* showed more disturbances in neonatal sleep, indicating that sleep patterns provide insight into the neurobehavior of newborn withdrawal (26).”

Please revise lines 221-224. Why would an infant with a longer gestation spend longer time in the hospital compared to an infant born at a shorter gestation (ie - 23 weeks gestation). Also, the correlation between dose exposure and severity of withdrawal continues to be debated.

Authors: We agree that drawing conclusions from this literature may be difficult; however, we feel that it is important information to outline in the background. We have taken steps to clarify that hospital stay was associated with higher doses of opioid treatment, but that length of NOWS treatment was associated with gestational age. In other words, a 23-week preterm infant may be in the hospital longer, but may undergo treatment for NOWS for a shorter period than infants with longer gestations. We provide a theory on why this may be occurring. We also acknowledge that dose and severity of withdrawal is a topic that continues to be debated.

“Newborns exposed to higher doses of methadone *in utero* were more likely to be treated for NOWS and to spend longer in the hospital than newborns exposed to lower doses (50); however, the effect of opioid dose on the severity of withdrawal continues to be debated. Further, newborns were treated for NOWS longer if they had a longer gestation (37-42 weeks treated for an average of 39.5 days; 33-36 weeks treated for an average of 20 days; 23-32 weeks treated for an average of 10 days). These findings may be due to the high permeability of the placenta in the late third trimester (51) or that current assessments are not geared toward assessing NOWS in preterm infants who may display differences in neurological development and, therefore, withdrawal signs at birth.”

Methods - when is the cry recording started? The methods states it continues during the NNNS-II but doesn't say the start. Does the infant keep the HRV leads on during the NNNS-II assessment? Is this felt to have any impact on the results of the NNNS?

Authors: The cry recording starts at the beginning of the NNNS-II assessment. We stop the recording at the end of the NNNS-II and then restart the cry recorder. We leave the cry recorder on for the remaining duration of the infant’s hospital stay. This results in distinct recordings of the infant’s cry during the NNNS-II and after the NNNS-II.

“The cry recording begins during the NNNS-II, is stopped after the assessment concludes, and then is restarted to record continuously for the length of hospital stay.”

The HRV leads are left on the infant’s chest during the NNNS-II assessment to avoid additional stress to the infant prior to the NNNS-II. The HRV leads are not felt to have any impact on the results of the NNNS-II.

“Then we measure infant heartrate, placing three electrodes on the upper (negative) and lower (positive) chest around the infant’s heart, and on the right lower side of the chest (neutral) and are left on the infant for the full newborn visit.”

Was IRB approval obtained - this was found later in the manuscript at the end of the methods? At one point consented is mentioned, but IRB approval is not included. Please move the IRB information, with the date range of enrollment (not given) to the beginning of the methods.

Authors: We moved the IRB information to the beginning of the Methods sections and included the dates of enrollment/data collection.

“This study was approved by the Institutional Review Board at the University of Utah on September 14^th^, 2019 (00124221) and Women and Infants Hospital on February 13^th^, 2020 (1479081-3). Written consent was obtained for all participants. Enrollment and data collection began in 2019 and will be completed in 2025.”

Line 297 - "caregiver changes may be an indicator of problem behavior." Do you have evidence to support this is true in this population? This is a high risk population, and so caregiver changes may occur due to caregiver indications. Consider removing this sentence.

Authors: We thank the reviewer for the feedback. Given the robust evidence that caregiver changes are related to increased risk for problems behavior, we theorized that caregiver changes may have a detrimental impact on development. We now cite this literature.

Fisher PA, Stoolmiller M, Mannering AM, Takahashi A, Chamberlain P. Foster placement disruptions associated with problem behavior: Mitigating a threshold effect. J Consult Clin Psychol. 2011;79(4):481–7.

Bada HS, Langer J, Twomey J, Bursi C, Lagasse L, Bauer CR, et al. Importance of Stability of Early Living Arrangements on Behavior Outcomes of Children With and Without Prenatal Drug Exposure. J Dev Behav Pediatr [Internet]. 2008 Jun [cited 2024 Apr 14];29(3):173. Available from: https://journals.lww.com/jrnldbp/fulltext/2008/06000/importance_of_stability_of_early_living.5.aspx?casa_token=686Fig2bOS4AAAAA:aVWtBIgzo_M0AtjbgFr_b8

What was the timing of enrollment in the study? I ask given that Finnegan was used to assess withdrawal, and ESC is becoming the gold standard approach for assessment.

Authors: The enrollment of the study began in 2019. Some participants are assessed with the Eat, Sleep, Console (ESC), as the University of Utah Hospital implemented this assessment in the maternal newborn unit in 2023; however, the NWI is still used in other units (e.g., NICU). We now cite and report use of the ESC.

If the caregiver at the 6 month visit was not the birthing caregiver, what data was collected (if any)? Consider changing maternal measures to birthing caregiver measures.

Authors: We appreciate the reviewer for highlighting this point. If the caregiver at the 6-month visit is not the birthing parent, we collect all the same self-report measures from the primary caregiver; however, they complete a different version of the infant feeding survey (Infant Feeding CG), which has modified questions regarding breastfeeding.

“If the birthing parent is not available, the primary caregiver reports these measures to best reflect the environment of the child.”

Under the 6 month infant measures, please remove those items collected at 18 months, at that makes it confusing to readers. Include those items under the 18 month infant measures.

Authors: We apologize for the confusion. We assessed several measures at both 6 and 18 months, we know provide in depth information for each measure at the first age it is used and then list the measure at the remaining ages it is used, while referencing the previous section.

If the caregiver at the 18 month visit was not the birthing caregiver, what data was collected (if any)? Consider changing maternal measures to birthing caregiver measures

If the caregiver at the 18-month visit is not the birthing parent, we collect all the same self-report measures from the primary caregiver.

“If the birthing parent is not available, the primary caregiver reports these measures to best reflect the environment of the child.”

Reviewer #3: It is a carefully done piece of work. I am extremely satisfied with the content, structure, and scientific approach of this study protocol. I firmly believe that it is a great work with great public health significance.

Authors: We appreciate the reviewer’s time and the positive feedback.

7. PLOS authors have the option to publish the peer review history of their article ([what does this mean?](https://urldefense.com/v3/__https:/journals.plos.org/plosone/s/editorial-and-peer-review-process*loc-peer-review-history__;Iw!!OToaGQ!qPCw3_ZC9IXOckCyoGIyu8uNbaMpNvlu6HSo5mtqcWPut8o-UQc70dT8oMwdJ83V-Z5FlUGh1rd3UZGsLQpo$)). If published, this will include your full peer review and any attached files.


If you choose “no”, your identity will remain anonymous but your review may still be made public.


**Do you want your identity to be public for this peer review?** For information about this choice, including consent withdrawal, please see our [Privacy Policy](https://urldefense.com/v3/__https:/www.plos.org/privacy-policy__;!!OToaGQ!qPCw3_ZC9IXOckCyoGIyu8uNbaMpNvlu6HSo5mtqcWPut8o-UQc70dT8oMwdJ83V-Z5FlUGh1rd3Ud7p454Y$).

Reviewer #1: No

Reviewer #3: No

[NOTE: If reviewer comments were submitted as an attachment file, they will be attached to this email and accessible via the submission site. Please log into your account, locate the manuscript record, and check for the action link "View Attachments". If this link does not appear, there are no attachment files.]

While revising your submission, please upload your figure files to the Preflight Analysis and Conversion Engine (PACE) digital diagnostic tool, [https://pacev2.apexcovantage.com/](https://urldefense.com/v3/__https:/pacev2.apexcovantage.com/__;!!OToaGQ!qPCw3_ZC9IXOckCyoGIyu8uNbaMpNvlu6HSo5mtqcWPut8o-UQc70dT8oMwdJ83V-Z5FlUGh1rd3UQeqqmfK$). PACE helps ensure that figures meet PLOS requirements. To use PACE, you must first register as a user. Registration is free. Then, login and navigate to the UPLOAD tab, where you will find detailed instructions on how to use the tool. If you encounter any issues or have any questions when using PACE, please email PLOS at [figures@plos.org](mailto:figures@plos.org). Please note that Supporting Information files do not need this step.
